# Supplementary material for: ASXLs binding to the PHD2/3 fingers of MLL4 provides a mechanism for the recruitment of BAP1 to active enhancers
Source: Nat Commun. 2024 Jun 7;15:4883. doi: 10.1038/s41467-024-49391-x (PMC11161652; doi:10.1038/s41467-024-49391-x)
Supplement: Supplementary file 1 — Supplementary Information [file 41467_2024_49391_MOESM1_ESM.pdf]

## Supplementary Information

# ASXLs binding to the PHD2/3 fingers of MLL4 provides a mechanism for the recruitment of BAP1 to active enhancers

Yi Zhang et al.,

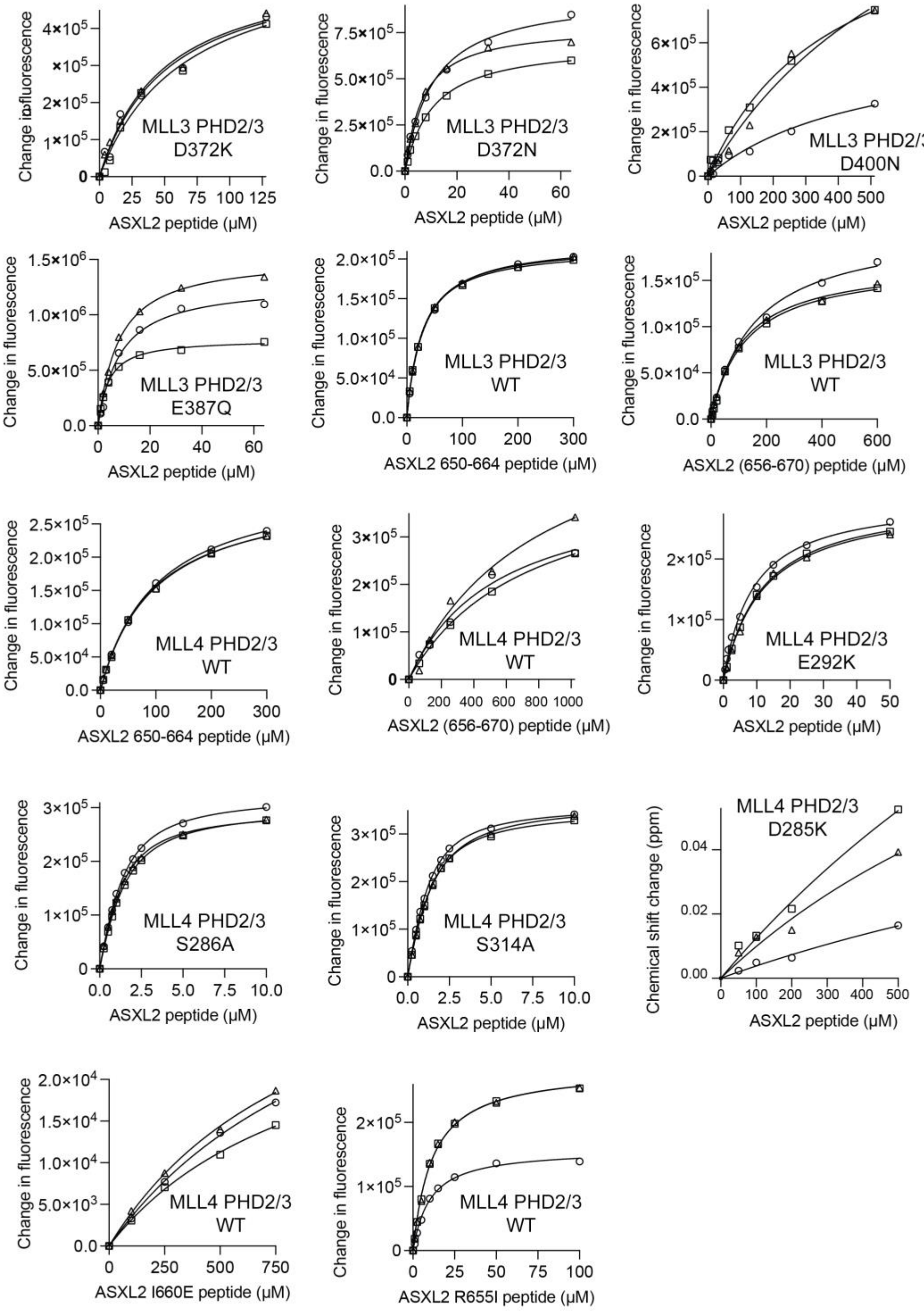


**Supplementary Figure 1. Measurements of binding affinities of WT and mutated MLL3$_{PHD2/3}$ and MLL4$_{PHD2/3}$ to the indicated WT and mutated ASXL2 peptides.** Binding curves used to determine the $K_d$ values by fluorescence spectroscopy and NMR. Related to Figures 1, 3 and 4.

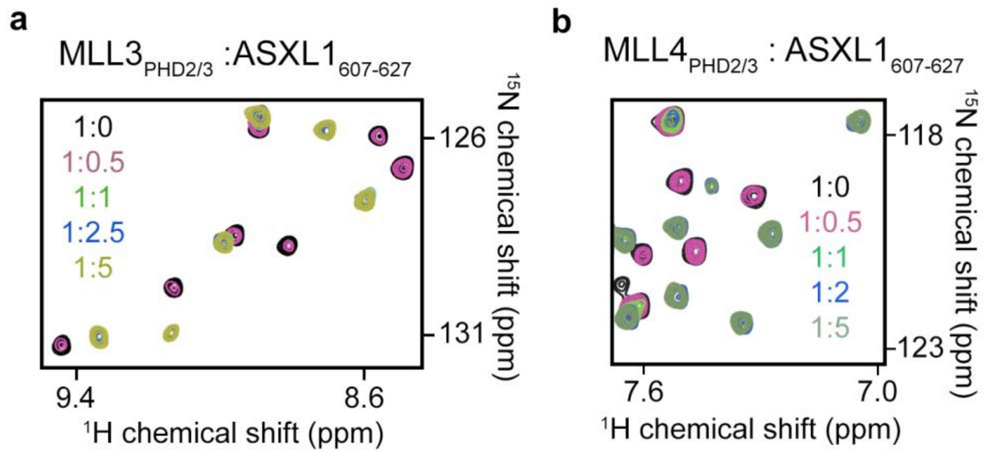


**Supplementary Figure 2. MLL3$_{PHD2/3}$ and MLL4$_{PHD2/3}$ interact with ASXL1.** (a, b) Superimposed $^{1}$H,$^{15}$N HSQC spectra of MLL3$_{PHD2/3}$ (a) and MLL4$_{PHD2/3}$ (b) collected upon titration with ASXL1 (aa 607-627 of ASXL1) peptide. Spectra are color coded according to the protein:peptide molar ratio. Related to Figure 1.

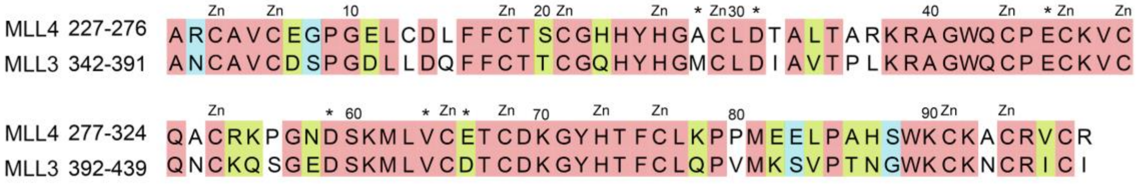


**Supplementary Figure 3. Alignment of the amino acid sequences of the PHD2/3 fingers from homologous MLL3 and MLL4.** Zinc coordinating (Zn) and described in the text (*) residues are indicated. Related to Figure 1.

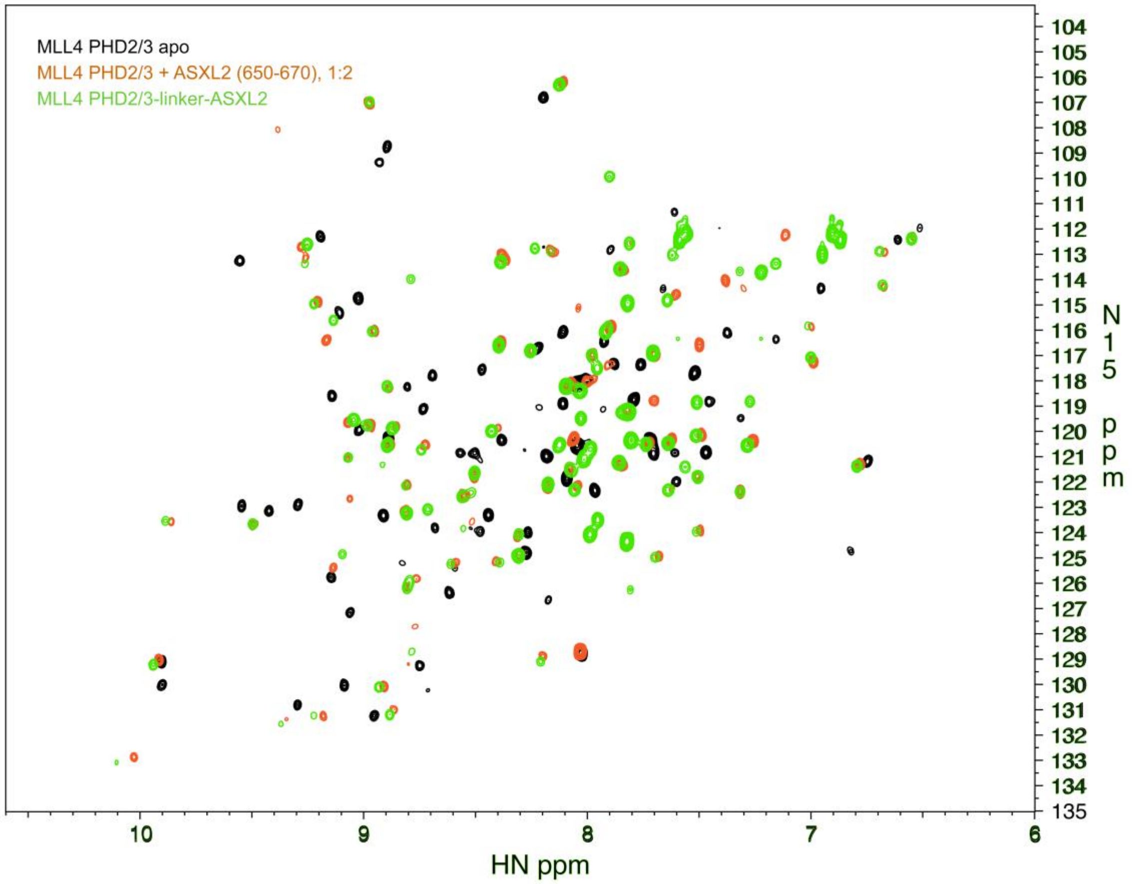


**Supplementary Figure 4. Analysis of the complex formation in MLL4$_{PHD2/3}$ constructs.** Superimposed $^{1}H$,$^{15}N$ HSQC spectra of MLL4$_{PHD2/3}$ in the apo-state (black), ASXL2 peptide-bound unlinked MLL4$_{PHD2/3}$ (red) and linked MLL4$_{PHD2/3}$-ASXL2 (green). Related to Figure 2.

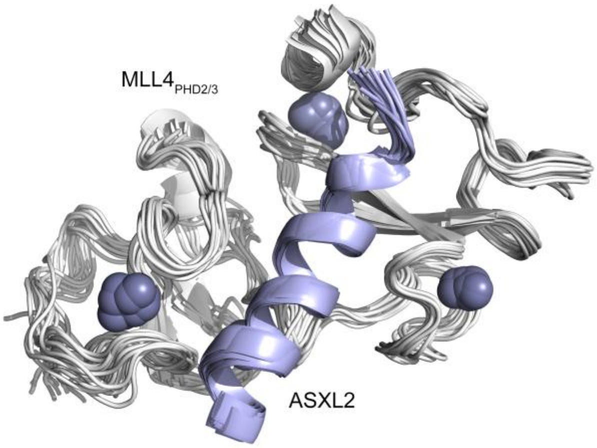


**Supplementary Figure 5.** Superimposition of 15 final NMR structures of MLL4$_{PHD2/3}$ in complex with ASXL2 (light blue). Related to Figure 2.

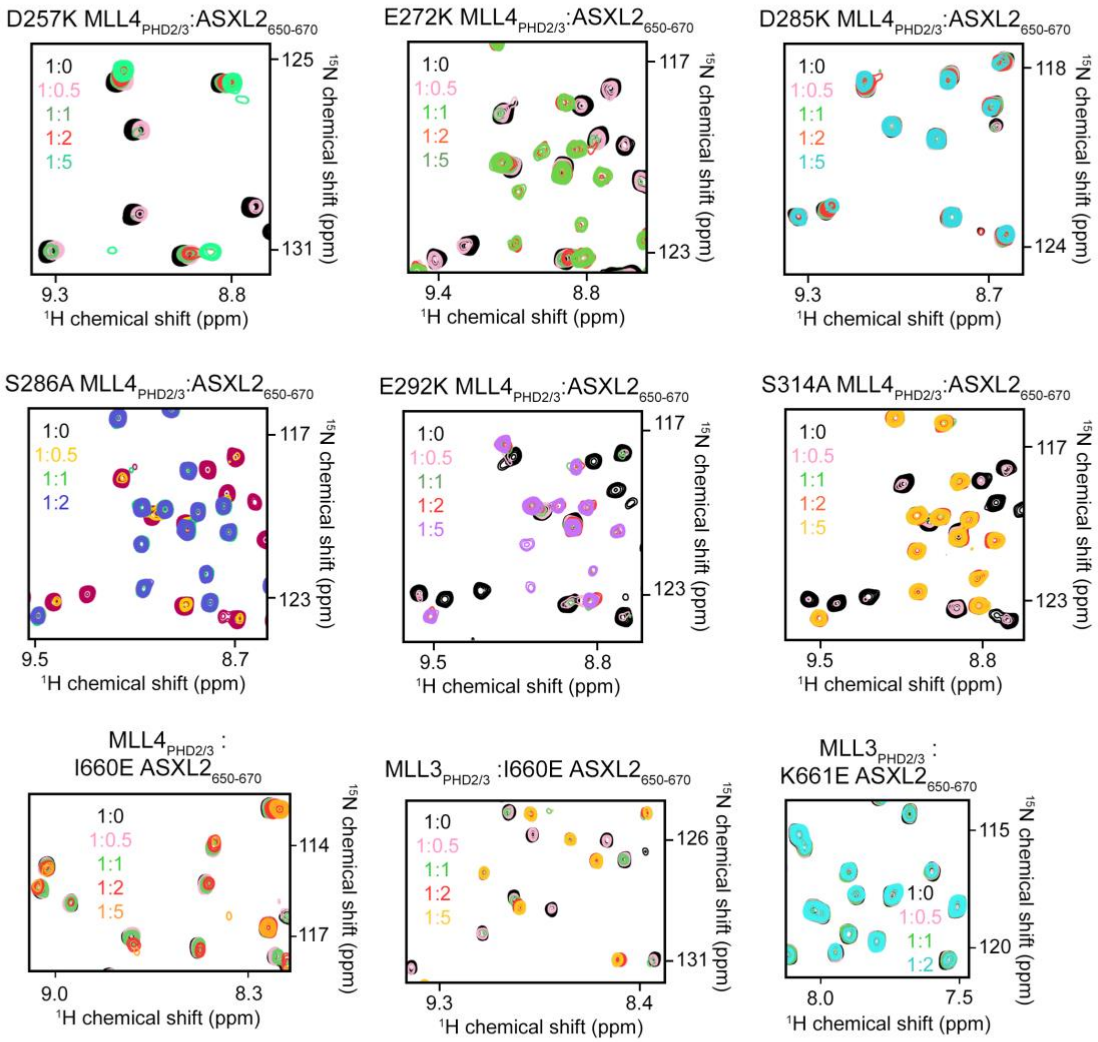


**Supplementary Figure 6. Interactions between the indicated MLL3/4$_{PHD2/3}$ constructs and ASXL2 peptides monitored by NMR.** Superimposed $^{1}H$,$^{15}N$ HSQC spectra of the MLL3/4$_{PHD2/3}$ constructs collected upon titration with the indicated peptides. Spectra are color coded according to the protein:peptide molar ratio. Related to Figures 3 and 4.

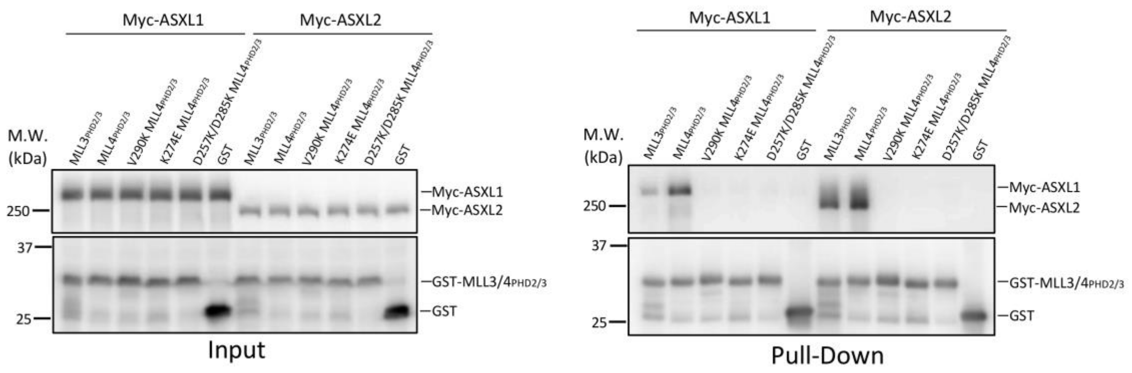


**Supplementary Figure 7. Human ASXL1 and ASXL2 interact with MLL3/4$_{PHD2/3}$.** Western blot analysis of ASXL1 and ASXL2, pulled down by recombinant wild-type or mutated GST-MLL3$_{PHD2/3}$ and GST-MLL4$_{PHD2/3}$. GST-MLL proteins were purified from bacteria and bound to GSH resin. Human Myc-ASXL1 and Myc-ASXL2 were expressed in HEK293FT cells. n=3 Related to Figure 3.

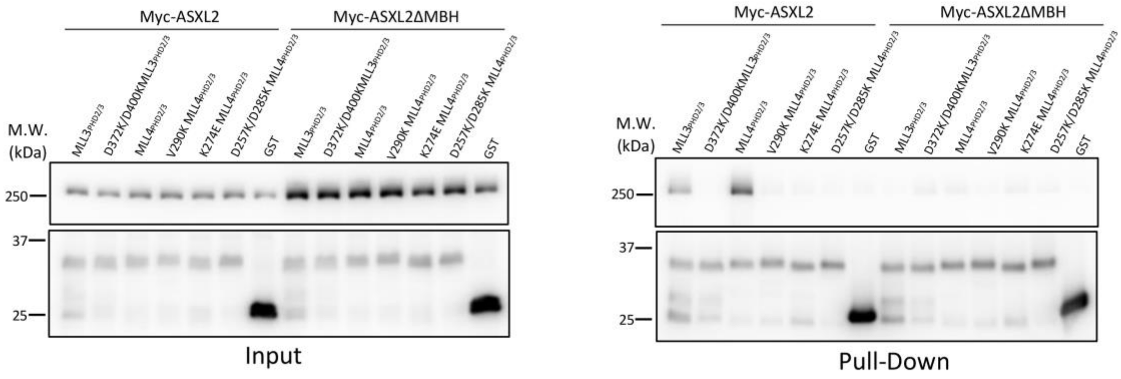


**Supplementary Figure 8. Human ASXL2 lacking the MLL-interacting motif (MBH) does not interact with $MLL3/4_{PHD2/3}$.** GST pull-down assay of recombinant GST-$MLL3_{PHD2/3}$ and GST-$MLL4_{PHD2/3}$ with human Myc-ASXL2 or Myc-ASXL2ΔMBH expressed in HEK293FT cells. Input and pull-down samples were analyzed by western blotting. Related to Figure 3.

a

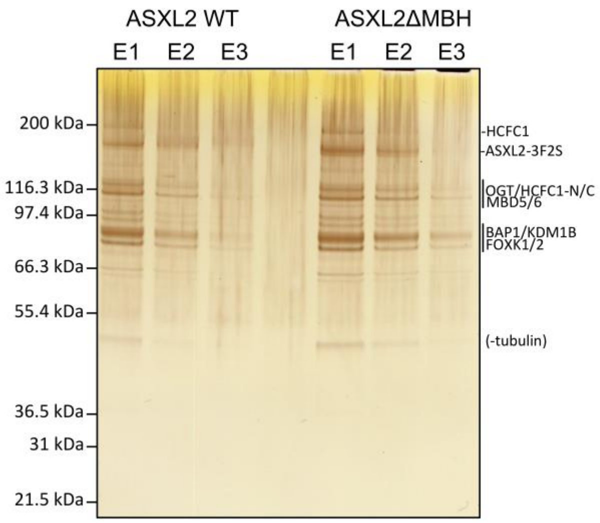


b

| | Total Spectral Counts | |
|---|---|---|
| Protein ID | ASXL2 WT | ASXL2ΔMBH |
| ASXL2 154kDa | 660 | 718 |
| HCFC1 209kDa | 476 | 433 |
| KDM1B/LSD2 92kDa | 315 | 291 |
| BAP1 80kDa | 235 | 234 |
| OGT 117kDa | 113 | 130 |
| FOXK1 75kDa | 52 | 52 |
| FOXK2 69kDa | 99 | 87 |
| MBD6 101kDa | 94 | 86 |
| MBD5 89kDa | 73 | 39 |
| YY1 45kDa | 2 | 0 |
| KMT2C/MLL3 | 34 | 0 |
| KMT2D/MLL4 | 4 | 0 |
| PAXIP1/PTIP | 11 | 0 |
| KDM6A/UTX | 11 | 0 |
| ASH2L | 10 | 0 |
| RBBP5 | 7 | 2 |
| NCOA6 | 3 | 0 |
| WDR5 | 6 | 5 |
| DPY30 | 0 | 0 |

**Supplementary Figure 9.** (a) Silver-stained gel of fractions obtained from the tandem affinity purification of WT ASXL2 and ASXL2 ΔMBH from K562 nuclear extracts. (b) Proteins identified by mass spectrometry proteomic analysis of tandem affinity purified fractions of from K562 cells expressing WT ASXL2 or ASXL2 ΔMBH. Subunits of the MLL3/4 complex are highlighted wheat, as in Figure 5b. Related to Figure 5.

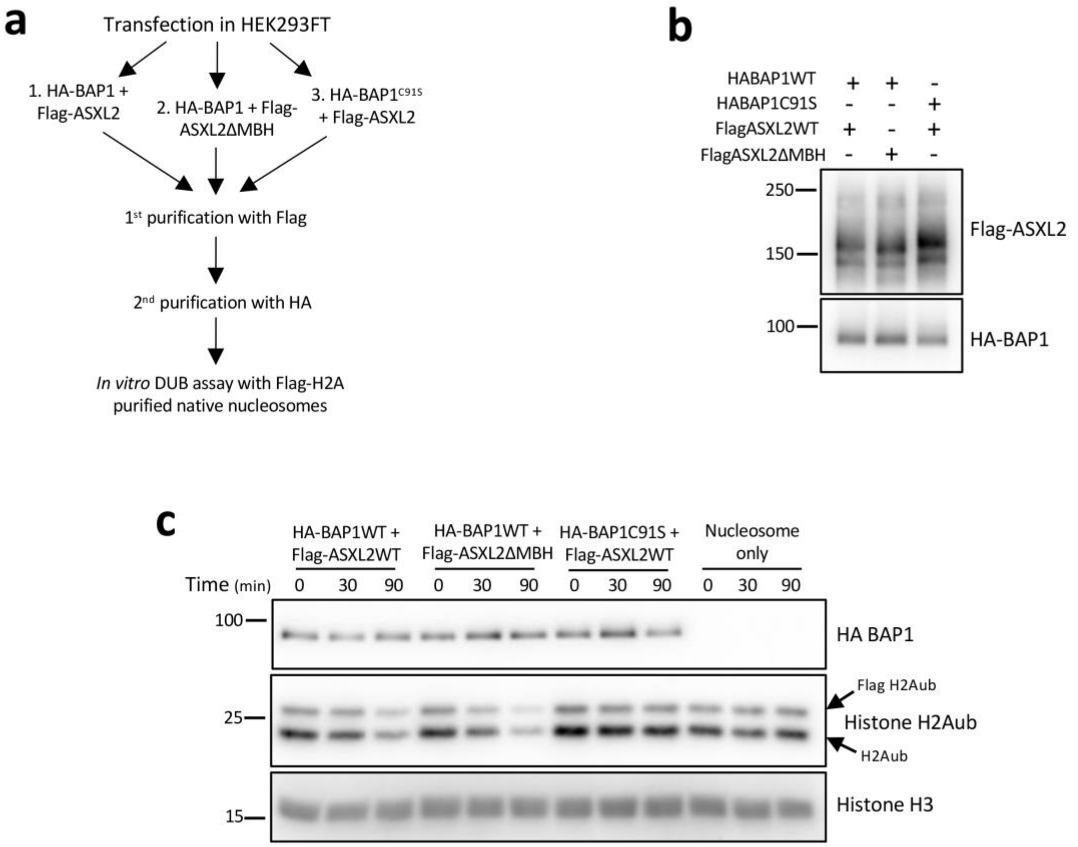


**Supplementary Figure 10. Loss of the MLL-interacting motif (MBH) does not affect BAP1/ASXL2 deubiquitinase activity toward H2AK119Ub *in vitro*.** (a, b) HEK293FT cells were co-transfected with wild-type or inactive (C91S) HA-BAP1 in combination with wild-type or ΔMBH Flag-ASXL2. BAP1/ASXL2 complexes were purified by tandem affinity purification (TAP) (a) and quantified by western blot (b). (c) *In vitro* deubiquitination assay was conducted using purified native nucleosomes with HA-BAP1/Flag-ASXL2, HA-BAP1/Flag-ASXL2ΔMBH or HA-BAP1C91S/Flag-ASXL2 complexes purified by TAP. Reactions were analyzed at the indicated times by western blot (n=2). Related to Figure 5.

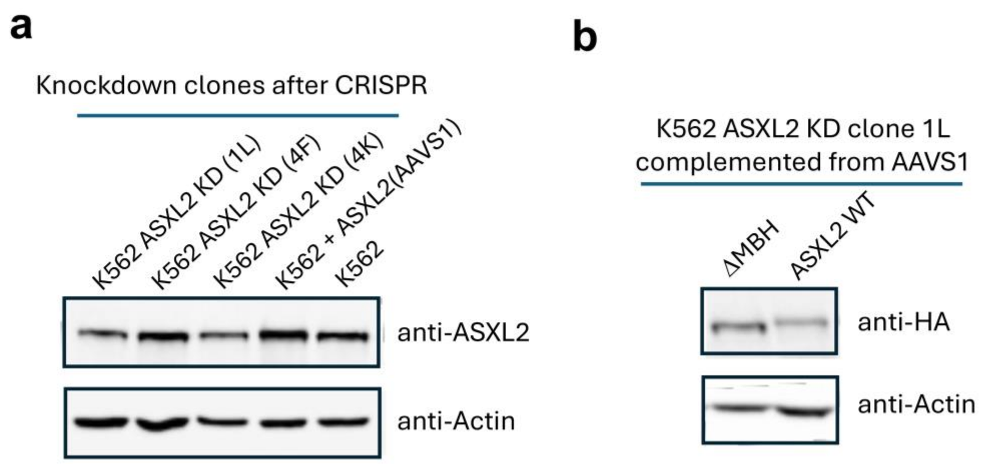


**Supplementary Figure 11. Selection of K562 clones with knocked down expression of endogenous ASXL2 and complementation with tagged WT and ΔMBH ASXL2 expressed form the AAVS1 safe harbor.** (a) Immunoblot of selected K562 cell clones after infection with lentivirus expressing Cas9 and gRNAs targeting the endo of ASXL2 exon 1. 20 mg of whole cell extracts are loaded and screened with anti-Asxl2 (Cell Signaling (E623X), CS71257, lot:1) and anti-Beta Actin signal (Thermo-Fisher AM4302, lot: 2666237) as loading control. Clone 1L was chosen as it shows the stronger knock down. (b) Clone 1L was used to stably transfect/express WT and ΔMBH ASXL2 from the AAVS1 locus. Anti-HA (Roche, anti-HA-HRP, clone 3F10, lot: 23551600) was used to detect expression for the transgenes after clonal selection. Related to Figure 5.

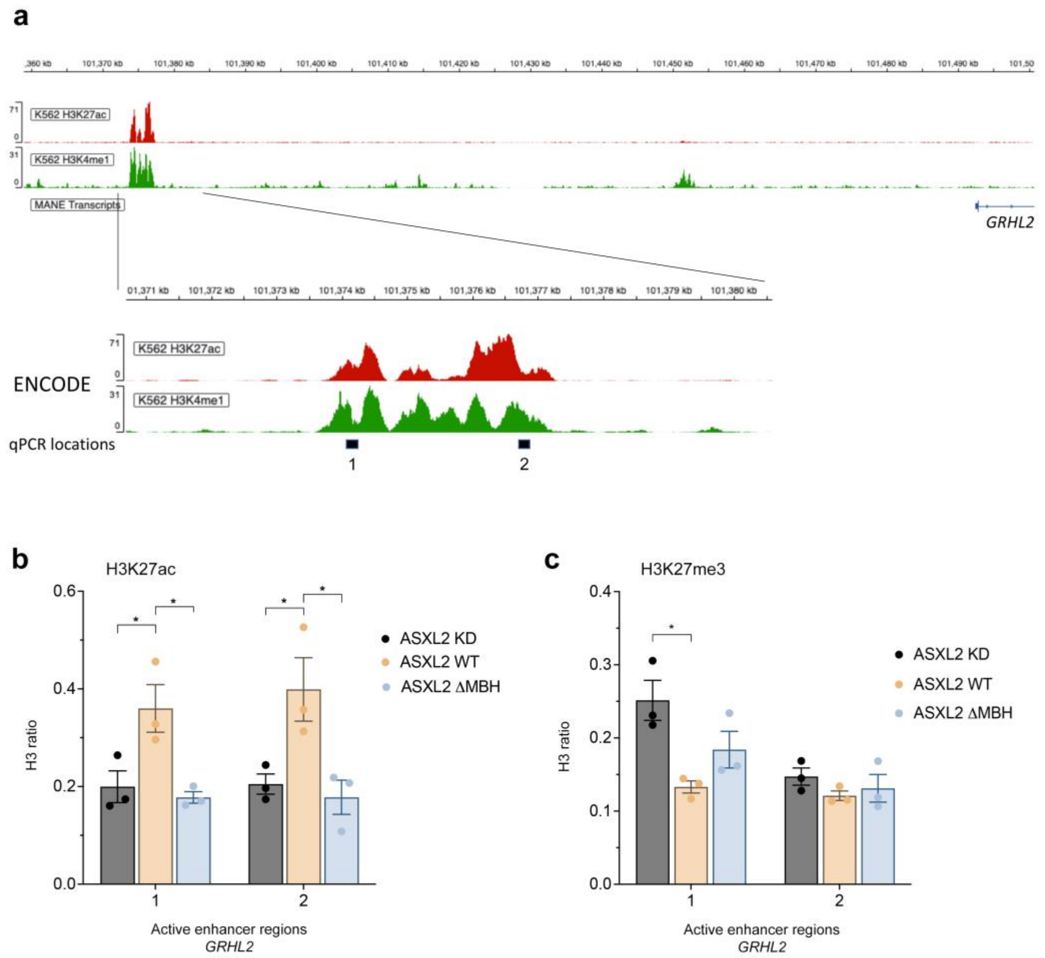


**Supplementary Figure 12. ChIP-qPCR of H3K27 acetylation/methylation at an active enhancer located on chromosome 8 upstream of the *GRHL2* gene.** (a) IGV window of a region of human chromosome 8 containing an active enhancer in K562 cells based on H3K27ac and H3K4me1 ChIP-seq profiles from the ENCODE project (tracks shown). Positions of amplicons used in ChIP-qPCR are shown in the zoom in window. (b, c) ChIP-qPCR analysis of H3K27ac (b) and H3K27me3 (c) in K562 ASXL2 KD cells complemented or not with WT or ΔMBH ASXL2 stably expressed from the *AAVS1* locus. The two regions indicated in (a) bordering the active enhancer upstream of the *GRHL2* gene are measured. Histone PTM levels were corrected for nucleosome occupancy (total H3 signal), presented as a ratio of IP/input (H3K27ac or me3/total H3). Data represent mean ± SEM from three biological replicates. Related to Figure 5.

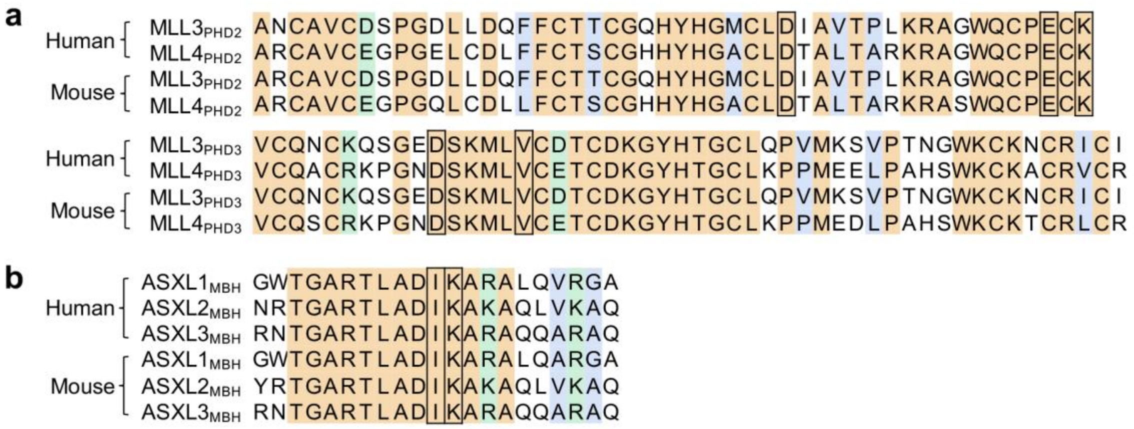

**Supplementary Figure 13.** Sequence alignment of human and mouse (a) $MLL3_{PHD2/3}$ and $MLL4_{PHD2/3}$ and (b) MBH of ASXL1/2/3. Key residues are outlined by rectangles. Related to Figure 6.

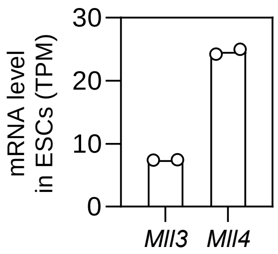


**Supplementary Figure 14. *Mll3* and *Mll4* mRNA levels in mouse ESCs.** Data from RNA-seq (GSE154475) are presented as dot plots (*n* = 2). Horizontal lines represent mean values. Related to Figure 6.

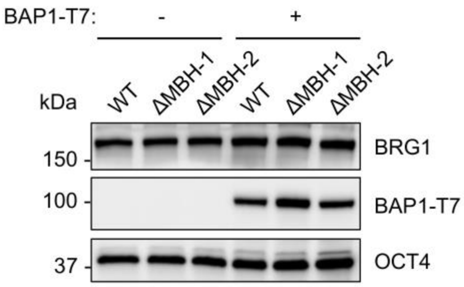


**Supplementary Figure 15. Immunoblotting of T7-tagged BAP1 (BAP1-T7).** Wild type (WT), ΔMBH-1 and ΔMBH-2 ESCs were infected with Doxycycline (Dox)-inducible lentiviral vector expressing BAP1-T7. Cells were treated with 1 µg/ml Dox to induce BAP1-T7 expression. Whole cell lysates were analyzed. BRG1 is shown as a loading control. Related to Figure 6.

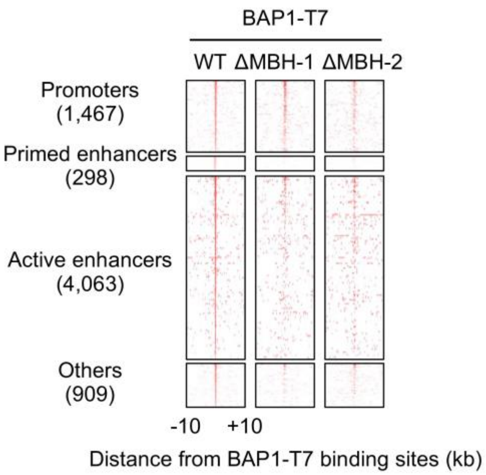


**Supplementary Figure 16.** Heat maps of BAP1-T7 occupancy on $BAP1^{+}$ $MLL4^{+}$ regions identified in Figure 7b. Related to Figure 7.

**Supplementary Table 1.** NMR and refinement statistics for the $MLL4_{PHD2/3}$-ASXL2 complex structure

| **$MLL4_{PHD2/3}$-ASXL2** | |
|---|---|
| **NMR distance and dihedral restraints** | |
| Distance constraints | |
| Total NOE | 2429 |
| Intra-residue | 799 |
| Inter-residue | |
| Sequential ($\lvert i - j \rvert = 1$) | 597 |
| Medium-range ($\lvert i - j \rvert \leq 4$) | 400 |
| Long-range ($\lvert i - j \rvert \geq 5$) | 633 |
| Hydrogen bonds | 24 |
| Total dihedral angle restraints | |
| $\phi$ | 39 |
| $\psi$ | 39 |
| | |
| **Structure statistics** | |
| Violations (mean and s.d.) | |
| Distance constraints (Å) | 0.023 ± 0.001 |
| Dihedral angle constraints (º) | 0.488 ± 0.107 |
| Max. distance constraint violation (Å) | 0.304 |
| Max. dihedral angle violation (º) | 4.882 |
| Deviations from idealized geometry | |
| Bond lengths (Å) | 0.004 ± 0.000 |
| Bond angles (º) | 0.638 ± 0.018 |
| Impropers (º) | 0.476 ± 0.021 |
| Average pairwise r.m.s. deviation** (Å) | |
| Heavy | 1.115 ± 0.137 |
| Backbone | 0.846 ± 0.128 |

**Pairwise r.m.s. deviation to mean structure was calculated among 15 refined structures. Residues 227-322 of MLL4 and 652-670 of ASXL2 were used. The percentage of residues in the most favored, additionally allowed, generously allowed and disallowed regions is 77.0, 19.4, 3.1 and 0.5, respectively.
