# Supplementary material for: ASXLs binding to the PHD2/3 fingers of MLL4 provides a mechanism for the recruitment of BAP1 to active enhancers
Source: Nat Commun. 2024 Jun 7;15:4883. doi: 10.1038/s41467-024-49391-x (PMC11161652; doi:10.1038/s41467-024-49391-x)
Supplement: Supplementary file 3 — Reporting Summary [file 41467_2024_49391_MOESM3_ESM.pdf]

nature portfolio

Corresponding author(s): Tatiana Kutateladze, Kai Ge

Last updated by author(s): May 3, 2024

Reporting Summary

Nature Portfolio wishes to improve the reproducibility of the work that we publish. This form provides structure for consistency and transparency in reporting. For further information on Nature Portfolio policies, see our Editorial Policies and the Editorial Policy Checklist.

Statistics

For all statistical analyses, confirm that the following items are present in the figure legend, table legend, main text, or Methods section.

n/a	Confirmed	
☐	☒	The exact sample size (*n*) for each experimental group/condition, given as a discrete number and unit of measurement
☐	☒	A statement on whether measurements were taken from distinct samples or whether the same sample was measured repeatedly
☐	☒	The statistical test(s) used AND whether they are one- or two-sided<br>*Only common tests should be described solely by name; describe more complex techniques in the Methods section.*
☒	☐	A description of all covariates tested
☒	☐	A description of any assumptions or corrections, such as tests of normality and adjustment for multiple comparisons
☐	☒	A full description of the statistical parameters including central tendency (e.g. means) or other basic estimates (e.g. regression coefficient) AND variation (e.g. standard deviation) or associated estimates of uncertainty (e.g. confidence intervals)
☐	☒	For null hypothesis testing, the test statistic (e.g. *F*, *t*, *r*) with confidence intervals, effect sizes, degrees of freedom and *P* value noted<br>*Give P values as exact values whenever suitable.*
☒	☐	For Bayesian analysis, information on the choice of priors and Markov chain Monte Carlo settings
☒	☐	For hierarchical and complex designs, identification of the appropriate level for tests and full reporting of outcomes
☒	☐	Estimates of effect sizes (e.g. Cohen's *d*, Pearson's *r*), indicating how they were calculated

Our web collection on statistics for biologists contains articles on many of the points above.

Software and code

Policy information about availability of computer code

Data collection	NMR experiments were carried out at 298K on Bruker 600, Varian INOVA 600 and 900 MHz spectrometers at the UC Denver NMR Core facility.
Data analysis	CcpNmr Suite, Xplor-NIH, and other software listed in the Method section. Software for ChIP-seq analysis include bowtie2 (v2.3.4.1), SICER algorithm (v2), R (v4.3.1) and IGV (v2.16.2), GREAT v3, SeqPos listed in Method section.

For manuscripts utilizing custom algorithms or software that are central to the research but not yet described in published literature, software must be made available to editors and reviewers. We strongly encourage code deposition in a community repository (e.g. GitHub). See the Nature Portfolio guidelines for submitting code & software for further information.

Data

Policy information about availability of data

All manuscripts must include a data availability statement. This statement should provide the following information, where applicable:

- Accession codes, unique identifiers, or web links for publicly available datasets
- A description of any restrictions on data availability
- For clinical datasets or third party data, please ensure that the statement adheres to our policy

The atomic coordinates and NMR assignments of MLL4-PHD23 in complex with ASXL2 peptide have been deposited in the Protein Data Bank under the accession

codes 9ATN and BMRB entry 31041. All ChIP-Seq data sets described in the paper have been deposited in NCBI Gene Expression Omnibus accession number GSE248027. The mm10 was used as the mouse genome reference.

Research involving human participants, their data, or biological material

Policy information about studies with human participants or human data. See also policy information about sex, gender (identity/presentation), and sexual orientation and race, ethnicity and racism.

Reporting on sex and gender	*Use the terms sex (biological attribute) and gender (shaped by social and cultural circumstances) carefully in order to avoid confusing both terms. Indicate if findings apply to only one sex or gender; describe whether sex and gender were considered in study design; whether sex and/or gender was determined based on self-reporting or assigned and methods used. Provide in the source data disaggregated sex and gender data, where this information has been collected, and if consent has been obtained for sharing of individual-level data; provide overall numbers in this Reporting Summary. Please state if this information has not been collected. Report sex- and gender-based analyses where performed, justify reasons for lack of sex- and gender-based analysis.*
Reporting on race, ethnicity, or other socially relevant groupings	*Please specify the socially constructed or socially relevant categorization variable(s) used in your manuscript and explain why they were used. Please note that such variables should not be used as proxies for other socially constructed/relevant variables (for example, race or ethnicity should not be used as a proxy for socioeconomic status). Provide clear definitions of the relevant terms used, how they were provided (by the participants/respondents, the researchers, or third parties), and the method(s) used to classify people into the different categories (e.g. self-report, census or administrative data, social media data, etc.) Please provide details about how you controlled for confounding variables in your analyses.*
Population characteristics	*Describe the covariate-relevant population characteristics of the human research participants (e.g. age, genotypic information, past and current diagnosis and treatment categories). If you filled out the behavioural & social sciences study design questions and have nothing to add here, write "See above."*
Recruitment	*Describe how participants were recruited. Outline any potential self-selection bias or other biases that may be present and how these are likely to impact results.*
Ethics oversight	*Identify the organization(s) that approved the study protocol.*

Note that full information on the approval of the study protocol must also be provided in the manuscript.

Field-specific reporting

Please select the one below that is the best fit for your research. If you are not sure, read the appropriate sections before making your selection.

☒ Life sciences ☐ Behavioural & social sciences ☐ Ecological, evolutionary & environmental sciences

For a reference copy of the document with all sections, see nature.com/documents/nr-reporting-summary-flat.pdf

Life sciences study design

All studies must disclose on these points even when the disclosure is negative.

Sample size	No statistical method was used to predetermine the sample size. The data size was determined based on published literature and previous experience.
Data exclusions	No data were excluded.
Replication	All results from representative experiments (such as micrographs and immunoblots) were collected at least twice independently. Generally, replications represented high correlations.
Randomization	No randomization.
Blinding	Investigators were not blinded in in vitro or cell culture experiments, because in vitro and cell culture experiments require frequent intervention by investigators to maintain materials with different feature and cell lines with different genotypes.

Reporting for specific materials, systems and methods

We require information from authors about some types of materials, experimental systems and methods used in many studies. Here, indicate whether each material, system or method listed is relevant to your study. If you are not sure if a list item applies to your research, read the appropriate section before selecting a response.

Materials & experimental systems

n/a	Involved in the study	
☐	☒	Antibodies
☐	☒	Eukaryotic cell lines
☒	☐	Palaeontology and archaeology
☒	☐	Animals and other organisms
☒	☐	Clinical data
☒	☐	Dual use research of concern
☒	☐	Plants

Methods

n/a	Involved in the study	
☐	☒	ChIP-seq
☒	☐	Flow cytometry
☒	☐	MRI-based neuroimaging

Antibodies

Antibodies used	Primary antibodies: Anti-BRG1 (ab110641, 1:5000 for WB), anti-OCT4 (ab19857, 1:1000 for WB) and anti-H3K27ac (ab4729, 2 μg for each ChIP) were from Abcam. Anti-T7 (D9E1X, 13246, 1:1000 for WB, 4 μg for each ChIP), AntiH2AK119ub (D27C4), Anti H3 (1B1B2) and Anti-ASXL2 (E6Z3X) were from Cell Signaling Technology. Anti-RbBP5 (A300-109A, 1:5000 for WB) was from Bethyl Laboratories. Anti-H3K4me1 (13-0040, 2 μg for each ChIP) was from EpiCypher. Anti-UTX (#2, 1:3000 for WB), Anti-HA, Anti-Myc and anti-MLL4 (#3, 1:3000 for WB, 2 μg for each IP, 8 μg for each ChIP) antibodies were homemade.<br>Secondary antibodies: Anti-Rabbit IgG, HRP-linked (7074, 1:2000 or WB) and anti-Mouse IgG, HRP-linked (7076, 1:2000 or WB) were from Cell Signaling Technology.
Validation	All antibodies were validated by the manufacturer or used in previous studies.<br>Anti-BRG1 (ab110641, Abcam): validated by manufacturer, https://www.abcam.com/brg1-antibody-epncir111a-ab110641<br>Anti-OCT4(ab19857, Abcam): validated by manufacturer, https://www.abcam.com/products/primary-antibodies/oct4-antibody-ab19857<br>Anti-H3K27ac (ab4729, Abcam): validated by manufacturer, https://www.abcam.com/histone-h3-acetyl-k27-antibody-chip-grade-ab4729<br>Anti-T7 tag (13246S, Cell Signaling Technology): https://pubmed.ncbi.nlm.nih.gov/33888555/<br>Anti-RBBP5 (A300-109A, Bethyl Laboratories): validated by manufacturer, https://www.fortislife.com/products/primary-antibodies/rabbit-anti-rbbp5-antibody/BETHYL-A300-109<br>Anti-H3K4me1 (13-0040, EpiCypher): validated by manufacturer, https://www.epicypher.com/products/antibodies/snap-chip-certified-antibodies/histone-h3k4me1-antibody-snap-chip-certified<br>Anti-UTX (#2, home-made): https://pubmed.ncbi.nlm.nih.gov/18003914/<br>Anti-MLL4 (#3, home-made): https://pubmed.ncbi.nlm.nih.gov/17500065/

Eukaryotic cell lines

Policy information about cell lines and Sex and Gender in Research

Cell line source(s)	Mouse embryonic stem cell lines used in this study: V6.5 wild type ESC was a gift from Dr. Chengyu Liu in NIH. K562 cell line used in this study was purchased from ATCC.
Authentication	None of the cell lines used were authenticated.
Mycoplasma contamination	Cell lines were validated as no mycoplasma contamination using Universal Mycoplasma Detection Kit (30-1012K) from ATCC.
Commonly misidentified lines<br>(See ICLAC register)	No commonly misidentified cell lines were used.

Plants

Seed stocks	*Report on the source of all seed stocks or other plant material used. If applicable, state the seed stock centre and catalogue number. If plant specimens were collected from the field, describe the collection location, date and sampling procedures.*
Novel plant genotypes	*Describe the methods by which all novel plant genotypes were produced. This includes those generated by transgenic approaches, gene editing, chemical/radiation-based mutagenesis and hybridization. For transgenic lines, describe the transformation method, the number of independent lines analyzed and the generation upon which experiments were performed. For gene-edited lines, describe the editor used, the endogenous sequence targeted for editing, the targeting guide RNA sequence (if applicable) and how the editor was applied.*
Authentication	*Describe any authentication procedures for each seed stock used or novel genotype generated. Describe any experiments used to assess the effect of a mutation and, where applicable, how potential secondary effects (e.g. second site T-DNA insertions, mosiacism, off-target gene editing) were examined.*

ChIP-seq

Data deposition

☒ Confirm that both raw and final processed data have been deposited in a public database such as GEO.

☒ Confirm that you have deposited or provided access to graph files (e.g. BED files) for the called peaks.

Data access links<br>*May remain private before publication.*	https://www.ncbi.nlm.nih.gov/geo/query/acc.cgi?acc=GSE248027
Files in database submission	WT-10_BAP1-3xT7_input_R1_001.fastq.gz<br>WT-10_BAP1-3xT7_input_R2_001.fastq.gz<br>AsxlMBH-2_BAP1-3xT7_input_R1_001.fastq.gz<br>AsxlMBH-2_BAP1-3xT7_input_R2_001.fastq.gz<br>AsxlMBH-27_BAP1-3xT7_input_R1_001.fastq.gz<br>AsxlMBH-27_BAP1-3xT7_input_R2_001.fastq.gz<br>WT-10_BAP1-3xT7_T7_R1_001.fastq.gz<br>WT-10_BAP1-3xT7_T7_R2_001.fastq.gz<br>AsxlMBH-2_BAP1-3xT7_T7_R1_001.fastq.gz<br>AsxlMBH-2_BAP1-3xT7_T7_R2_001.fastq.gz<br>AsxlMBH-27_BAP1-3xT7_T7_R1_001.fastq.gz<br>AsxlMBH-27_BAP1-3xT7_T7_R2_001.fastq.gz<br>WT-10_BAP1-3xT7_MLL4_R1_001.fastq.gz<br>WT-10_BAP1-3xT7_MLL4_R2_001.fastq.gz<br>AsxlMBH-2_BAP1-3xT7_MLL4_R1_001.fastq.gz<br>AsxlMBH-2_BAP1-3xT7_MLL4_R2_001.fastq.gz<br>AsxlMBH-27_BAP1-3xT7_MLL4_R1_001.fastq.gz<br>AsxlMBH-27_BAP1-3xT7_MLL4_R2_001.fastq.gz<br>WT-10_BAP1-3xT7_H3K4me1_R1_001.fastq.gz<br>WT-10_BAP1-3xT7_H3K4me1_R2_001.fastq.gz<br>AsxlMBH-2_BAP1-3xT7_H3K4me1_R1_001.fastq.gz<br>AsxlMBH-2_BAP1-3xT7_H3K4me1_R2_001.fastq.gz<br>AsxlMBH-27_BAP1-3xT7_H3K4me1_R1_001.fastq.gz<br>AsxlMBH-27_BAP1-3xT7_H3K4me1_R2_001.fastq.gz<br>WT-10_BAP1-3xT7_H3K27ac_R1_001.fastq.gz<br>WT-10_BAP1-3xT7_H3K27ac_R2_001.fastq.gz<br>AsxlMBH-2_BAP1-3xT7_H3K27ac_R1_001.fastq.gz<br>AsxlMBH-2_BAP1-3xT7_H3K27ac_R2_001.fastq.gz<br>AsxlMBH-27_BAP1-3xT7_H3K27ac_R1_001.fastq.gz<br>AsxlMBH-27_BAP1-3xT7_H3K27ac_R2_001.fastq.gz<br><br>WT-10_BAP1-3xT7_T7_sorted-W50-G50-FDR0.001-islandfiltered-normalized.wig<br>AsxlMBH-2_BAP1-3xT7_T7_sorted-W50-G50-FDR0.001-islandfiltered-normalized.wig<br>AsxlMBH-27_BAP1-3xT7_T7_sorted-W50-G50-FDR0.001-islandfiltered-normalized.wig<br>WT-10_BAP1-3xT7_MLL4_sorted-W50-G50-FDR1e-10-islandfiltered-normalized.wig<br>AsxlMBH-2_BAP1-3xT7_MLL4_sorted-W50-G50-FDR1e-10-islandfiltered-normalized.wig<br>AsxlMBH-27_BAP1-3xT7_MLL4_sorted-W50-G50-FDR1e-10-islandfiltered-normalized.wig<br>WT-10_BAP1-3xT7_H3K4me1_sorted-W200-G200-FDR0.001-islandfiltered-normalized.wig<br>AsxlMBH-2_BAP1-3xT7_H3K4me1_sorted-W200-G200-FDR0.001-islandfiltered-normalized.wig<br>AsxlMBH-27_BAP1-3xT7_H3K4me1_sorted-W200-G200-FDR0.001-islandfiltered-normalized.wig<br>WT-10_BAP1-3xT7_H3K27ac_sorted-W200-G200-FDR0.001-islandfiltered-normalized.wig<br>AsxlMBH-2_BAP1-3xT7_H3K27ac_sorted-W200-G200-FDR0.001-islandfiltered-normalized.wig<br>AsxlMBH-27_BAP1-3xT7_H3K27ac_sorted-W200-G200-FDR0.001-islandfiltered-normalized.wig
Genome browser session<br>(e.g. UCSC)	No longer applicable

Methodology

Replicates	ChIP-Seq data for two independent mutant cell lines are from single experiment.
Sequencing depth	Each library contains at least 20 million reads.
Antibodies	Anti-H3K4me1 (13-0040) was from EpiCypher. Anti-H3K27ac (ab4729) was from Abcam. Anti-T7 tag (13246S) was from Cell Signaling Technology. Anti-MLL4 was home-made. Spike-in antibody (61686) and chromatin (53083) were from Active Motif.
Peak calling parameters	For ChIP-Seq of histone modifications (H3K4me1 and H3K27ac), the window size of 200 bp, the gap size of 200 bp, and the false discovery rate (FDR) threshold of 1E-3 were used. For ChIP-Seq of MLL4, the window size of 50 bp, the gap size of 50 bp, and the FDR threshold of 1E-10 were used. For ChIP-Seq of T7, the window size of 50 bp, the gap size of 50 bp, and the FDR threshold of 1E-3 were used.
Data quality	For ChIP-Seq of histone modifications (H3K4me1 and H3K27ac) and T7 tag, peaks with the false discovery rate (FDR) < 1E-3 were included in the data analysis. For ChIP-Seq of MLL4 peaks with FDR < 1E-10 were included in the data analysis.

Software	Software for ChIP-Seq data processing and analysis include bowtie2 (v2.3.4.1), SICER algorithm (v2), R (v4.3.1) and IGV (v2.16.2).
